# Comprehensive CT Angiography Assessment of Internal Thoracic Artery Morphometry, Anatomical Relationships, and Perforating Branches

**DOI:** 10.3390/biomedicines14081863

**Published:** 2026-08-20

**Authors:** Selma Cansu Bayrak, Keziban Karacan, Alper Karacan, Mehtap Erdogan

**Affiliations:** 1 Department of Anatomy, Faculty of Medicine, Sakarya University, 54050 Sakarya, Turkey; scansubayrak96@gmail.com (S.C.B.); kkaracan@sakarya.edu.tr (K.K.); 2 Department of Radiology, Faculty of Medicine, Sakarya University, 54050 Sakarya, Turkey; alperkaracan@sakarya.edu.tr

**Keywords:** internal thoracic artery, morphometry, computed tomography angiography, intercostal space, vascular anatomy

## Abstract

**Background and Objectives:** The internal thoracic artery (ITA) is widely used in coronary artery bypass grafting and reconstructive procedures due to its favorable anatomical characteristics. This study aimed to comprehensively evaluate the morphometric features of the ITA using computed tomography angiography and to investigate variations according to sex and anatomical side. **Materials and Methods:** In this retrospective cross-sectional study, thoracic computed tomography angiography images of 72 individuals were analyzed. Bilateral measurements of ITA length, diameter at different intercostal levels, anatomical relationships, and perforating branches were recorded. **Results:** The mean ITA length was slightly greater on the left side (18.6 ± 2.7 cm) compared to the right (18.3 ± 2.3 cm), without significant sex differences. A progressive decrease in arterial diameter from proximal to distal segments was observed. Statistically significant sex-related differences in diameter were identified, particularly at the 3rd and 4th intercostal spaces (*p* < 0.05). Distances to adjacent anatomical structures also varied significantly between sexes. Perforating branches were most frequently detected at the 4th intercostal space. **Conclusions:** The ITA demonstrates notable morphometric variability depending on sex and anatomical side. These findings provide anatomical reference data that may contribute to individualized evaluation of the ITA.

## 1. Introduction

The internal thoracic artery (ITA), also known as the internal mammary artery, is one of the major arteries that originates from the proximal portion of the subclavian artery and plays a key role in the blood supply to the anterior thoracic wall. It typically originates from the subclavian artery approximately 2 cm superior to the sternal end of the clavicle and opposite the thyrocervical trunk [1,2,3,4]. The ITA enters the thoracic cavity through the superior thoracic aperture and runs inferiorly along the inner surface of both anterior chest walls, approximately 1 cm lateral to the lateral margin of the sternum. As it travels, it gives off perforating branches in the intercostal spaces, supplying blood to the breast tissue and a significant portion of the anterior chest wall [2,4]. The artery typically branches into its terminal branches—the superior epigastric artery and the musculophrenic artery—at the level of the sixth or seventh intercostal space. The superior epigastric artery contributes to the blood supply of the anterior abdominal wall by anastomosing with the inferior epigastric artery, while the musculophrenic artery runs along the ribs and plays a role in the vascularization of the diaphragm [4,5]. Recent review studies have demonstrated that there are clinically significant variations in the origin, course, and branching patterns of the internal thoracic artery, and that these variations must be taken into account in surgical planning [6].

Although the origin and course of the ITA are described in a specific order in anatomy textbooks, various variations regarding the origin, course, and branching of this artery have been reported in the literature. It is known that the ITA most commonly originates from the prescalene segment of the subclavian artery. However, its origin from the extrascalene (third) portion of the subclavian artery is a rare variation, reported in 0.5–1.0% of anatomical studies and approximately 1.5% of angiographic studies [6,7]. In a case report by Omar and colleagues, a rare variation was described in which the ITA originates from the third segment of the subclavian artery on both sides [3]. In addition, variations in which the ITA originates from different vascular structures have also been reported in the literature. Jiang and colleagues reported that the left internal thoracic artery arises from a common trunk with the ipsilateral vertebral artery and the thyrocervical trunk [8]. Similarly, Cigali and colleagues have described a variation in which the left ITA originates from the thyrocervical trunk. These variations underscore the importance of a detailed anatomical assessment of the ITA in the context of surgical and interventional procedures. Similar variability has also been reported in other major arterial systems evaluated by CTA, highlighting the broad spectrum of vascular anatomical differences that may have clinical significance [9,10].

The clinical significance of the ITA is particularly evident in the fields of thoracic surgery, cardiovascular surgery, and reconstructive surgery. With the development of coronary artery bypass grafting (CABG) surgery in the 20th century, the ITA has become one of the most frequently used graft vessels due to its high patency rate and long-term success outcomes [11,12,13]. In addition, the ITA is widely used as an important recipient vessel in breast surgery and reconstructive surgery. It is frequently preferred, particularly in autologous breast reconstruction, due to its suitable diameter, surgical accessibility, and relatively well-preserved anatomical position within the thorax [6,14]. The central location of the ITA within the thoracic cavity contributes to this artery being less affected by radiation therapy compared to other arteries in the region and offers an advantage in terms of reconstructive surgery.

Given its wide range of applications in surgical procedures, a detailed understanding of the morphometric characteristics of the ITA is of great importance. ITA morphometry encompasses the artery’s origin from the subclavian artery, its length, diameter, relationship with adjacent anatomical structures, and potential variations. Although there are various studies in the literature on the length, diameter, or specific anatomical relationships of the ITA, the number of studies that evaluate these morphometric parameters within a comprehensive framework is limited. Studies based on computed tomography angiography have shown that the diameter, length, and branching characteristics of the internal thoracic artery can be reliably assessed and play an important role in identifying individual variations [15]. In addition, computed tomography angiography (CTA) has been widely used in the evaluation of vascular anatomy and anomalies in different regions of the body, providing high-resolution imaging and detailed morphometric analysis. Previous studies have demonstrated its effectiveness in identifying variations in coronary arteries, aortic arch branching patterns, and abdominal vascular structures, further supporting its reliability in anatomical research [9,16,17].

Therefore, the aim of our study was to evaluate the morphometric characteristics of the internal thoracic artery—such as length, diameter, and variations—using computed tomography angiography images and to compare the obtained data with respect to gender. It is anticipated that the findings from this study will contribute to clinical planning in the fields of thoracic surgery, reconstructive surgery, and interventional radiology. This study was conducted based on the hypothesis that ITA morphometric characteristics would exhibit measurable differences depending on sex and anatomical side.

## 2. Materials and Methods

This study was approved by the Sakarya University Faculty of Medicine Non-Interventional Ethics Committee (Approval No: 333, Date: 31 October 2023) and was conducted in accordance with the principles of the Declaration of Helsinki.

### 2.1. Study Design and Sample

This study was designed as a retrospective, cross-sectional study. The study analyzed chest computed tomography angiography (CTA) images acquired at Sakarya University Training and Research Hospital. A total of 72 individuals (37 women, 35 men) aged 18–69 were included in the study.

### 2.2. Inclusion and Exclusion Criteria

Individuals with high-quality, evaluable CT images of the thoracic region were included in the study. Individuals with poor image quality, prior thoracic surgery, vascular pathology, or anatomical distortion were excluded.

### 2.3. Imaging and Measurement Method

All images were obtained from the hospital’s archiving system and evaluated using appropriate imaging software. CTA is a well-established imaging modality that allows accurate visualization of vascular structures and has been successfully used in detecting both congenital and acquired vascular anomalies in various anatomical regions [9,16]. All measurements were performed in collaboration with an experienced radiologist. To assess the reliability of the measurements, randomly selected cases were re-evaluated by the same observer four weeks later, and intra-observer agreement was assessed. The internal thoracic artery (ITA) was examined bilaterally.

All CT angiography images were acquired using a 64-slice multidetector CT scanner (Aquilion 64, Toshiba, Tokyo, Japan). Imaging parameters were set as follows: 120 kV tube voltage, 120–380 mA tube current, and 1 mm slice thickness. The images were evaluated using the Aquarius 3D Workstation (TeraRecon, Durham, NC, USA).

All morphometric measurements were performed by a single researcher under the supervision of an experienced radiologist.

The CTA examinations included in this study were obtained for diagnostic or clinical evaluation purposes. Since this was a retrospective anatomical assessment study, the specific clinical indications of each examination were not analyzed. Individuals with vascular pathology, previous thoracic surgery, or anatomical distortion affecting ITA evaluation were excluded.

ITA morphometric measurements were performed using curved planar reconstruction (CPR) and multiplanar reconstruction (MPR) techniques to enable assessment along the course of the vessel and to reduce measurement errors caused by vessel bends. These reconstruction methods allowed evaluation of the ITA along its anatomical course.

**Figure 1:**
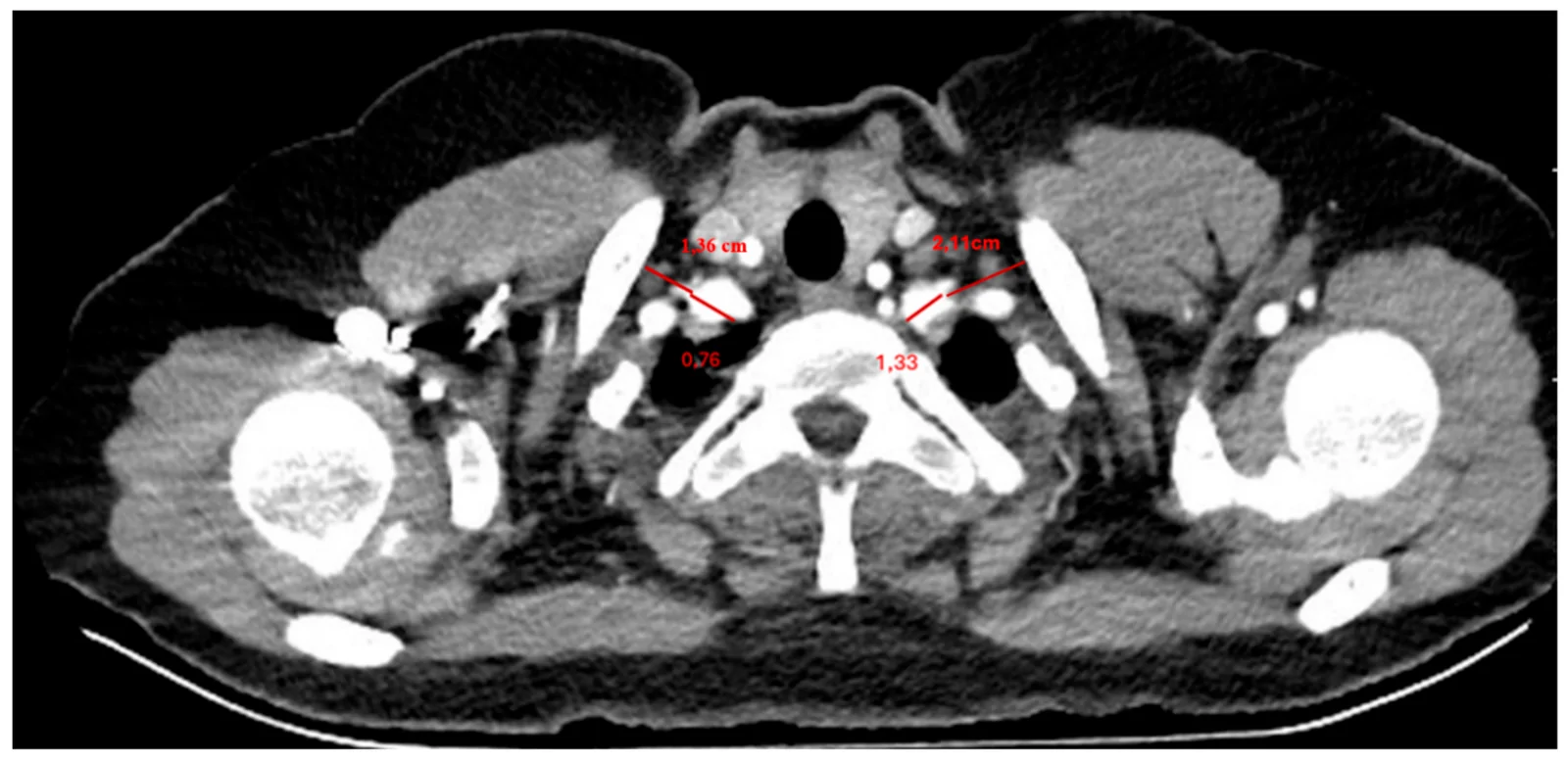
CTA-based visualization of the right internal thoracic artery origin. Coronal and sagittal oblique CPR images demonstrate the origin of the ITA from the subclavian artery and the measurement approach.

**Figure 2:**
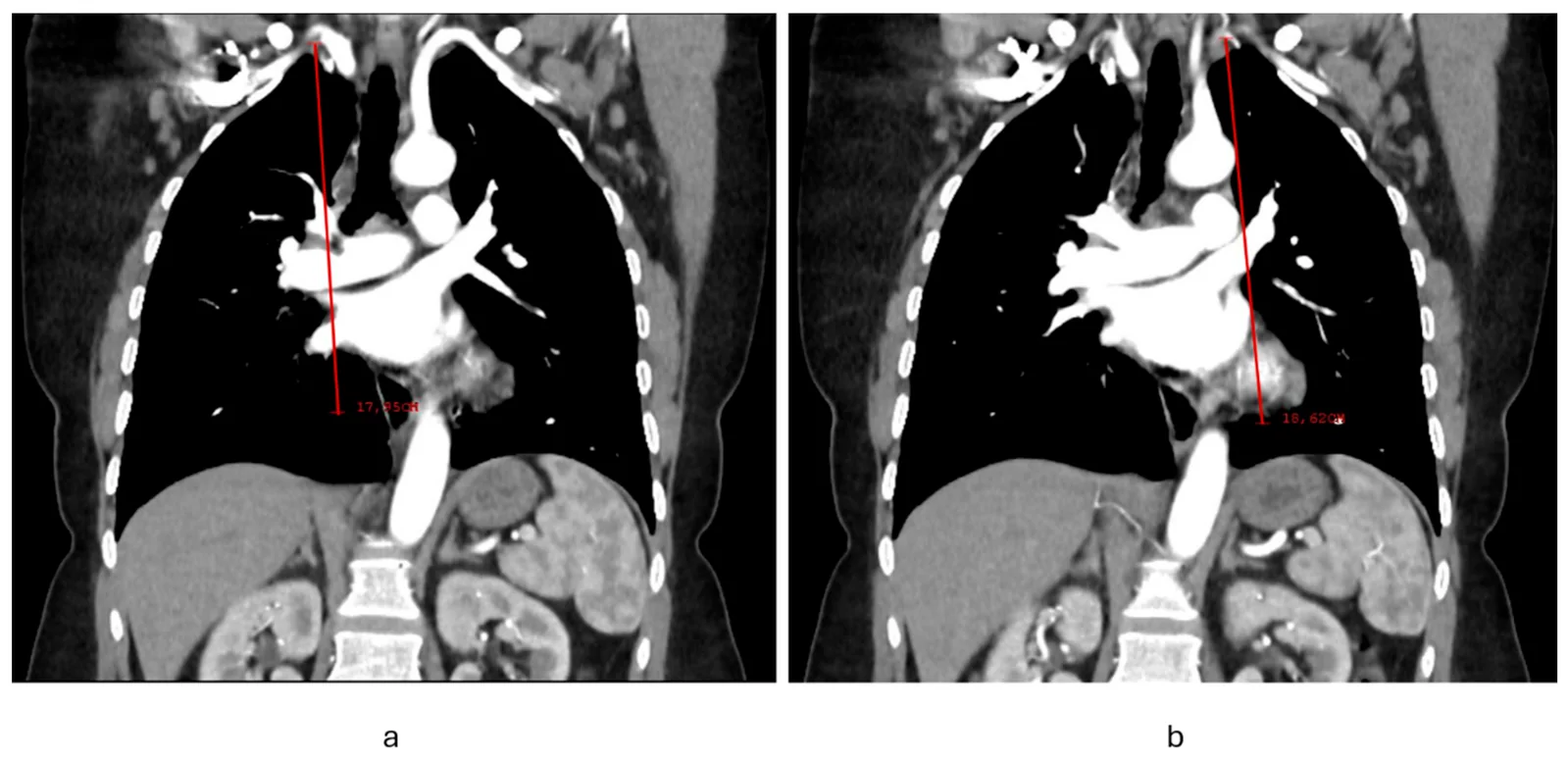
Measurement of ITA length. (**a**) Right, (**b**) left.

Measurements were obtained for the following parameters (Figure 1): total ITA length, diameter at the second, third, and fourth intercostal spaces, distance between the ITA and the sternum, and the presence of perforating branches. Diameter measurements were obtained at the widest cross-sectional lumen of the artery, whereas length measurements were performed between the arterial origin and terminal point. All measurements were recorded in millimeters (mm). ITA length measurements are illustrated in Figure 2.

### 2.4. Statistical Analysis

The data were analyzed using SPSS 25.0 (IBM Corp., Armonk, NY, USA). Continuous variables are expressed as mean ± standard deviation (SD), while categorical variables are presented as counts and percentages (%). The normality of data distribution was assessed using skewness and kurtosis values. Values within the range of −1.5 to +1.5 were accepted as indicating normal distribution, as suggested by Tabachnick and Fidell. For comparisons between groups, the independent samples *t*-test or Mann–Whitney U test was used as appropriate. Paired samples *t*-test was used for dependent variables. The chi-square test was applied for categorical variables. A *p*-value of <0.05 was considered statistically significant. Effect sizes were calculated to assess the magnitude of the observed differences in comparisons deemed statistically significant. To reduce the risk of error arising from multiple comparisons, adjusted *p*-values were considered where appropriate. A post hoc power analysis demonstrated a statistical power of 84% for the study. Because comparisons within each results table formed a related family of tests, the Benjamini–Hochberg false discovery rate (FDR) procedure was applied within each table to control for multiple comparisons, and both raw and FDR-adjusted *p*-values are reported. For normally distributed continuous variables, effect size was expressed as Cohen’s d; for non-normally distributed variables compared with the Mann–Whitney U test, the rank-biserial correlation (r) was used; for categorical comparisons (chi-square/Fisher’s exact test), Cramer’s V was used.

## 3. Results

A total of 72 individuals were evaluated in our study; 35 (48.6%) were male and 37 (51.4%) were female. A total of 144 images of the internal thoracic artery (ITA) were examined bilaterally in all cases.

When evaluating ITA length, the average length on the right side (right internal thoracic artery, RITA) was measured as 18.257 ± 2.303 cm, and on the left side (left internal thoracic artery, LITA) as 18.633 ± 2.656 cm. In general, the left ITA was observed to be longer than the right. When compared by gender, it was found that ITA lengths were higher in men than in women; however, this difference was not statistically significant (Table 1).

The termination of the ITA was observed at the level of the 6th or 7th intercostal space in all cases.

ITA diameter measurements were evaluated separately at the 1st–6th intercostal space (ICS) levels. In general, a decrease in ITA diameter from proximal to distal was observed. At the sixth intercostal space level, the RITA diameter was measured as 2.63 ± 0.42 mm, and the LITA diameter as 2.53 ± 0.40 mm. When analyzed by gender, the RITA diameter was found to be 2.55 ± 0.41 mm in women and 2.72 ± 0.41 mm in men. Statistically significant differences after FDR adjustment were observed at selected intercostal levels (Table 2).

**Figure 3:**
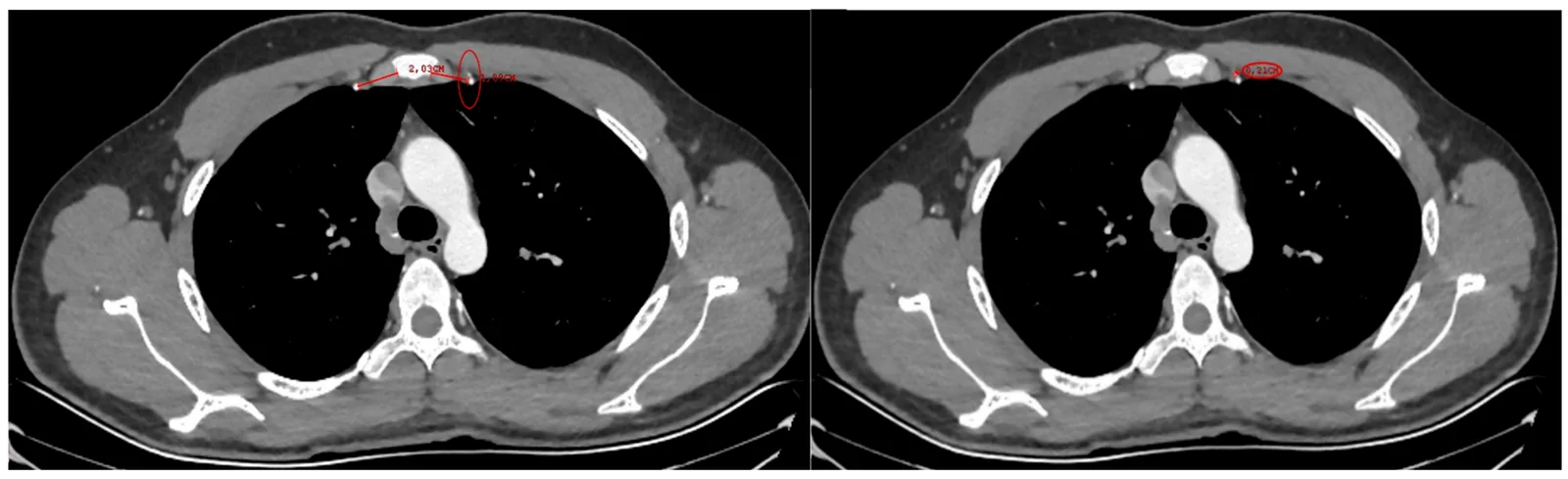
Measurement of ITA distance to the sternum and evaluation of perforating branches at the 3rd intercostal space.

The distances between the ITA and key anatomical structures were also measured and compared both overall and by gender. The data revealed that the distance between the ITA and the sternum and other adjacent anatomical structures varies among individuals (Table 3). In particular, the differences in diameter observed at the levels of the 3rd and 4th intercostal spaces are clinically significant because these regions are frequently used as recipient vessels in reconstructive surgery. The measurement of the distance to the sternum and evaluation of perforating branches are shown in Figure 3.

Distances between the ITA and adjacent anatomical structures showed variability among individuals. After FDR correction, the distance from the origin of the ITA to the clavicle remained significantly greater in females compared to males on both sides (right: *p* = 0.010; left: *p* = 0.001).

At the fourth intercostal level, the distance between the right ITA and pericardium was significantly higher in males (*p* = 0.024), whereas no significant difference was observed on the left side.

The distance between the ITA and the nipple was significantly greater in females for both sides (*p* < 0.001), which may be attributed to anatomical differences in breast tissue.

**Figure 4:**
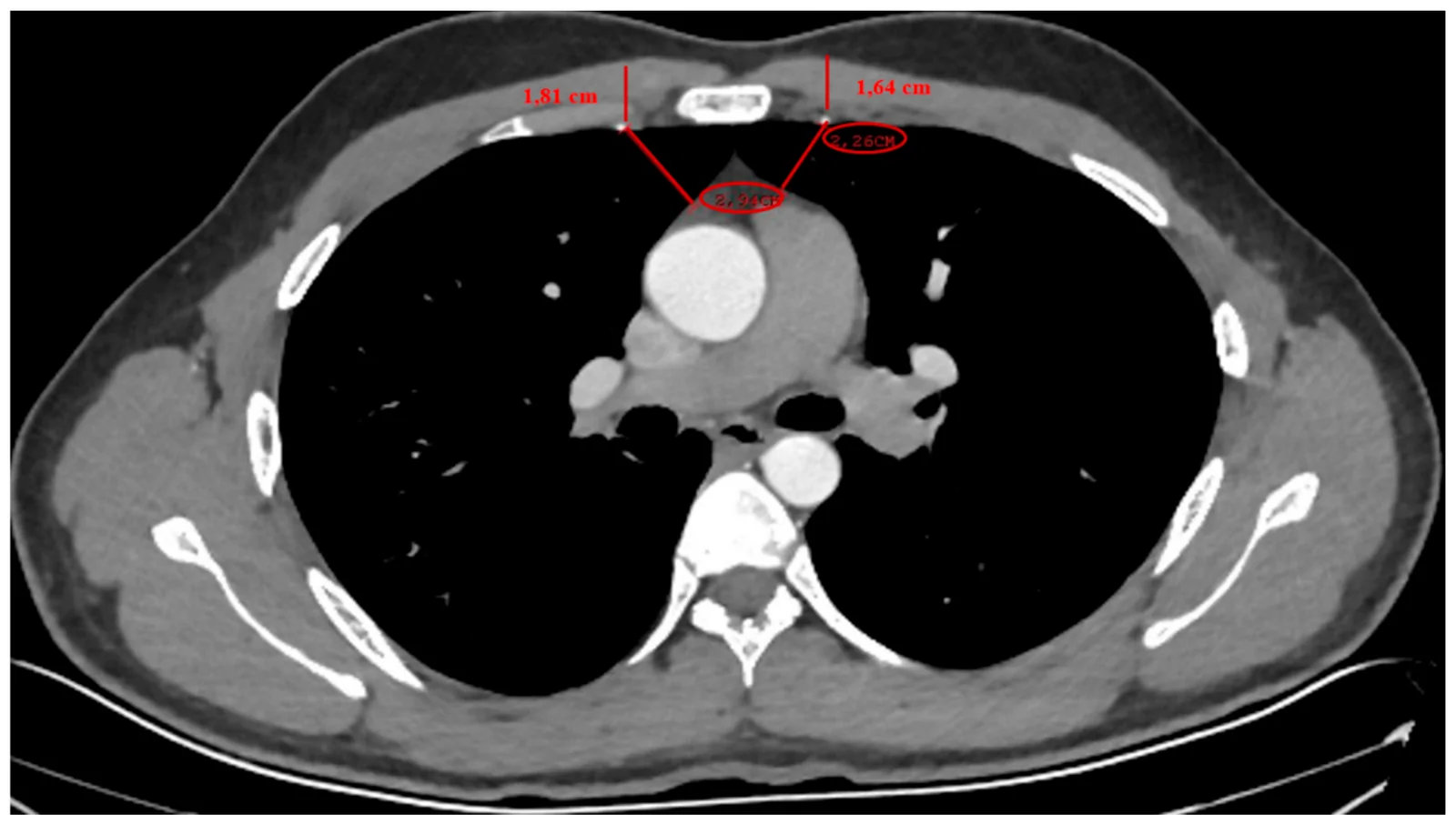
Measurement of ITA distance to the pericardium and thoracic wall at the 4th intercostal space.

Similarly, the distance between the ITA and thoracic wall was significantly greater in males on both sides (*p* < 0.001). Measurements related to pericardium and thoracic wall distances are illustrated in Figure 4.

The presence of perforating branches was assessed at the levels of the 1st through 5th intercostal spaces. It was determined that, in both female and male participants, perforating branches were most frequently observed at the level of the 4th intercostal space for both the right and left ITA. It was found that the incidence rates of perforating branches varied according to intercostal level and gender (Table 4).

Overall, the findings indicate that the ITA can vary both between sides and by gender in terms of length, diameter, anatomical relationships, and the distribution of perforating branches.

## 4. Discussion

To our knowledge, this study provides a comprehensive evaluation by simultaneously assessing the length, diameter, perforator distribution, and anatomical relationships of the ITA. In the present study, morphometric characteristics of the internal thoracic artery (ITA) were examined using computed tomography angiography images, and comparisons were made between genders and sides. The key findings of our study indicate that there may be interindividual variability in the length, diameter, distribution of perforating branches, and anatomical relationships of the ITA.

Although no significant difference was found between the right and left arteries in terms of ITA length, the left ITA was observed to be longer on average than the right. This finding is consistent with previous reports [6,7]. The absence of a significant difference in length between genders supports the notion that morphometric variations are primarily due to individual differences. The literature also emphasizes that interindividual variability in the anatomical characteristics of the ITA is clinically significant [6]. In line with these findings, previous CTA-based studies have also demonstrated significant anatomical variations in different vascular territories, including the subclavian artery and aortic arch, reinforcing the importance of preoperative vascular assessment [9,10].

In ITA diameter measurements, it was found that the diameter at certain intercostal space levels was significantly higher in males than in females. Statistically significant differences after FDR correction were observed at selected intercostal space levels. These results are consistent with data previously reported by [18,19]. Furthermore, studies based on computed tomography angiography have shown that the diameter of the ITA varies across intercostal levels and decreases from proximal to distal segments [20]. In addition, it has been reported that the distribution of perforating branches may vary among individuals [15]. Although the absolute differences in vessel diameter were relatively small, these minor differences may still be clinically relevant because appropriate vessel caliber matching may facilitate vascular anastomosis, particularly in microsurgical procedures and recipient vessel selection.

With regard to the presence of perforating vessels, it has been found that in both female and male individuals, perforating vessels are most commonly located at the level of the 4th intercostal space. This finding is consistent with studies by Fansa et al. (2013) [14], which emphasize the clinical importance of the distribution of perforating vessels for the selection of recipient vessels in autologous breast reconstruction. Studies on the localization of perforating vessels suggest that prior knowledge of these structures during surgical planning may reduce the risk of complications [21]. Similarly, detailed vascular imaging plays an important role in identifying anatomical variations that could affect surgical planning and in minimizing unexpected technical challenges that may arise during surgery [22].

The distances between the ITA and the sternum and other anatomical structures are of critical importance in terms of surgical access and flap planning. The measurements obtained in our study are consistent with classical anatomical values reported in the literature [23]. However, the variability observed among individuals necessitates careful preoperative assessment and intraoperative attention. The “1-2-3 rule,” which describes the expected location of the internal mammary vessels approximately 1 cm, 2 cm, and 3 cm lateral to the sternal border at the first, second, and third intercostal spaces, respectively, serves as a practical anatomical guide during recipient vessel exposure [24]. These anatomical landmarks may also facilitate surgical exposure and vessel identification during procedures in which the internal thoracic artery is harvested or exposed, including coronary artery bypass grafting (CABG) [25]. The distance measurements obtained in our study are consistent with these anatomical principles.

The clinical significance of the internal thoracic artery (ITA) is particularly evident in the fields of cardiovascular and reconstructive surgery. Current consensus reports emphasize that the ITA is one of the most reliable grafts with a high long-term patency rate in coronary artery bypass surgery [26]. Furthermore, recent meta-analyses support the notion that the use of bilateral internal thoracic arteries may offer advantages in terms of long-term clinical outcomes [27] Additionally, it has been reported that different harvesting techniques for the internal thoracic artery (skeletonized and pedicled) may influence morphological and clinical outcomes [28]. In this context, morphometric data obtained through CT angiography can enable a detailed preoperative assessment of the length, diameter, and anatomical course of the internal thoracic artery, as well as its relationships with surrounding anatomical structures. This information may be useful for the surgeon in selecting an appropriate graft, planning the technique for preparing a skeletonized or pedicled graft, and anticipating potential technical difficulties, particularly in cases where the use of bilateral internal thoracic arteries is being considered. With the advancements in surgical techniques today, the importance of the internal thoracic artery continues to grow, particularly in hybrid and advanced revascularization approaches.

The ITA’s central location within the thoracic cavity and its greater resistance to the effects of radiation therapy compared to other arteries are factors that enhance the reliability of this vessel in reconstructive surgery. This characteristic offers a clinical advantage in selecting the ITA as the recipient vessel, particularly in patients scheduled for autologous free-flap breast reconstruction following mastectomy.

Overall, consistent with data in the literature, our study demonstrates that there are individual, sex- and side-dependent variations in the morphometric characteristics of the ITA. These findings may provide reference anatomical data for the individual anatomical assessment of the ITA prior to surgical planning. Supporting this approach, recent literature based on CTA emphasizes that individualized vascular mapping is essential due to the wide variability observed in arterial anatomy across different regions of the body [9,10,16]. These findings may support anatomical evaluation before surgical procedures.

Although surgical outcomes were not directly evaluated in this study, it is believed that the morphometric findings obtained via CT angiography may contribute to preoperative surgical planning. Understanding individual variations in the diameter and length of the internal thoracic artery and its relationships with surrounding anatomical structures may help determine the most appropriate graft strategy, particularly in coronary artery bypass grafting (CABG) surgeries where the use of bilateral internal thoracic arteries or skeletonized harvesting techniques is planned. Similarly, detailed anatomical information regarding the distribution of perforating branches and the location of recipient vessels can facilitate the selection of an appropriate recipient vessel in microvascular breast reconstruction, contribute to safer surgical dissection, and reduce anatomical uncertainties that may be encountered during the operation. However, the findings of this study should be interpreted as anatomical reference data to support surgical planning, rather than as data predicting surgical outcomes. Therefore, in future prospective, multicenter studies, correlating the morphometric findings obtained via CT angiography with intraoperative findings, graft patency, and postoperative clinical outcomes will more comprehensively demonstrate their clinical applicability and translational value.

These findings may be particularly relevant for preoperative planning in microsurgical breast reconstruction, where accurate localization and diameter assessment of recipient vessels are critical for flap survival.

## 5. Conclusions

This study provides a comprehensive evaluation of the internal thoracic artery by analyzing its length, diameter, perforator distribution, and anatomical relationships using computed tomography angiography. The findings demonstrate that morphometric characteristics of the ITA may vary according to side and sex, highlighting the importance of individualized anatomical assessment. These findings may provide valuable guidance for surgical planning by contributing to a more accurate preoperative anatomical assessment, particularly in coronary artery bypass grafting and reconstructive surgical procedures.

### Limitations

This study has several limitations. First, its retrospective design may introduce selection bias and limits control over imaging and measurement conditions. Second, the sample size (n = 72), although sufficient for statistical analysis, may not fully represent the general population. However, the post hoc power analysis conducted in our study showed that the sample size had sufficient statistical power (84%) to detect the differences observed in this cohort. Nevertheless, the findings should be interpreted as anatomical reference data specific to this study group, which was derived from a single-center cohort of the Turkish population, rather than normative population-based reference values. Third, the study was conducted in a single center, which may limit the generalizability of the findings. Additionally, the study population consisted solely of individuals from a Turkish population, and therefore, the results may not be directly applicable to other ethnic groups. Finally, although all morphometric measurements were performed by a single researcher under the supervision of an experienced radiologist, interobserver reliability could not be assessed, and potential measurement bias cannot be completely excluded. In addition, although CPR and MPR reconstructions were used for morphometric assessment, the absence of dedicated centerline lumen reconstruction may have affected measurement accuracy, particularly in tortuous arterial segments. Future studies employing dedicated centerline lumen reconstruction software may further improve the accuracy and reproducibility of morphometric assessments. Accordingly, future multicenter prospective studies involving larger sample sizes and ethnically diverse populations, together with the correlation of CT angiography-derived morphometric findings with intraoperative findings, graft patency, and postoperative clinical outcomes, are needed to further validate and strengthen the generalizability, clinical applicability, and translational value of our findings.

## Figures and Tables

**Table 1:** Distribution of ITA lengths by sex and side.

	Overall (n = 72)	Female (n = 37)	Male (n = 35)	*p*
	Mean ± SD	Mean ± SD	Mean ± SD	
Age	52.05 ± 11.66	56.32 ± 9.27	47.54 ± 12.34	
ITA Length Right	18.257 ± 2.303	18.013 ± 2.265	18.516 ± 2.347	0.359 FDR *p* = 0.258 r = 0.164
ITA Length Left	18.633 ± 2.656	18.287 ± 2.674	18.998 ± 2.624	0.259 FDR *p* = 0.258 d = −0.269

ITA: internal thoracic artery; FDR *p*: Benjamini–Hochberg false discovery rate-adjusted *p*-value; d: Cohen’s d (effect size for independent samples *t*-test); r: rank-biserial correlation (effect size for Mann–Whitney U test).

**Table 2:** Comparison of internal thoracic artery (ITA) and subclavian artery diameters by intercostal space and sex (mm).

Level	ICS	Side	Overall (n = 72) Mean ± SD (mm)	Female (n = 37) Mean ± SD (mm)	Male (n = 35) Mean ± SD (mm)	*p*
Subclavian artery (origin of ITA)	-	Right	12.36 ± 2.17	11.91 ± 2.04	12.84 ± 2.23	0.071 FDR *p* = 0.126 d = −0.432
	-	Left	10.00 ± 1.76	10.02 ± 1.69	9.98 ± 1.85	0.911 FDR *p* = 0.826 r = −0.031
ITA	1st	Right	3.56 ± 0.67	3.48 ± 0.62	3.66 ± 0.72	0.264 FDR *p* = 0.338 d = −0.266
		Left	3.47 ± 0.65	3.37 ± 0.68	3.57 ± 0.62	0.217 FDR *p* = 0.240 r = 0.188
ITA	2nd	Right	3.32 ± 0.69	3.27 ± 0.57	3.36 ± 0.80	0.573 FDR *p* = 0.622 d = −0.133
		Left	3.24 ± 0.62	3.11 ± 0.58	3.38 ± 0.64	0.071 FDR *p* = 0.126 d = −0.432
ITA	3rd	Right	2.91 ± 0.57	2.73 ± 0.51	3.10 ± 0.57	0.005 * FDR *p* = 0.034 * r = 0.374
		Left	2.96 ± 0.53	2.93 ± 0.53	2.98 ± 0.53	0.657 FDR *p* = 0.598 r = 0.090
ITA	4th	Right	2.86 ± 0.46	2.73 ± 0.40	3.01 ± 0.48	0.009 * FDR *p* = 0.034 * d = −0.631
		Left	2.81 ± 0.41	2.70 ± 0.37	2.92 ± 0.42	0.022 * FDR *p* = 0.052 d = −0.554
ITA	5th	Right	2.70 ± 0.38	2.59 ± 0.32	2.82 ± 0.41	0.011 * FDR *p* = 0.034 * r = 0.354
		Left	2.66 ± 0.37	2.56 ± 0.36	2.77 ± 0.40	0.018 * FDR *p* = 0.042 * r = 0.332
ITA	6th	Right	2.63 ± 0.42	2.55 ± 0.41	2.72 ± 0.41	0.084 FDR *p* = 0.131 d = −0.413
		Left	2.53 ± 0.40	2.41 ± 0.35	2.65 ± 0.41	0.008 * FDR *p* = 0.034 * d = −0.641

n: number of subjects, ICS: intercostal space, SD: standard deviation, ITA: internal thoracic artery, *: *p* < 0.05; FDR *p*: Benjamini–Hochberg false discovery rate-adjusted *p*-value; d: Cohen’s d (effect size for independent samples *t*-test); r: rank-biserial correlation (effect size for Mann–Whitney U test). Bold indicates statistically significant differences (*p* < 0.05).

**Table 3:** Distances from the origin of the ITA to anatomical structures (cm).

Landmark		Overall (n = 72)	Female (n = 37)	Male (n = 35)	*p*
		Mean ± SD	Mean ± SD	Mean ± SD	
**Clavicle**	**Right**	2.469 ± 0.795	2.701 ± 0.742	2.223 ± 0.784	0.010 * FDR *p* = 0.016 * d = 0.626
	**Left**	2.650 ± 0.785	2.930 ± 0.714	2.354 ± 0.755	0.001 * FDR *p* = 0.003 * d = 0.785
**Sternum (2nd ICS)**	**Right**	1.310 ± 0.407	1.415 ± 0.399	1.200 ± 0.366	0.024 * FDR *p* = 0.030 * d = 0.544
	**Left**	1.230 ± 0.415	1.273 ± 0.384	1.186 ± 0.447	0.380 FDR *p* = 0.382 d = 0.208
**Pericardium (4th ICS)**	**Right**	2.339 ± 0.886	2.111 ± 0.852	2.580 ± 0.868	0.024 * FDR *p* = 0.016 * r = 0.347
	**Left**	1.476 ± 0.930	1.358 ± 0.812	1.602 ± 1.037	0.270 FDR *p* = 0.304 d = −0.262
**Nipple (4th ICS)**	**Right**	8.300 ± 1.823	9.210 ± 1.777	7.338 ± 1.319	<0.001 * FDR *p* < 0.001 * d = 1.191
	**Left**	8.337 ± 1.962	9.245 ± 1.991	7.378 ± 1.412	<0.001 * FDR *p* < 0.001 * r = −0.544
**Thoracic wall (4th ICS)**	**Right**	1.656 ± 0.373	1.454 ± 0.277	1.869 ± 0.344	<0.001 * FDR *p* < 0.001 * d = −1.330
	**Left**	1.562 ± 0.336	1.408 ± 0.277	1.724 ± 0.320	<0.001 * FDR *p* < 0.001 * d = −1.054

SD: standard deviation, ITA: internal thoracic artery, ICS: intercostal space, *: *p* < 0.05. Bold indicates statistically significant differences (*p* < 0.05).

**Table 4:** Comparison of the Presence of Perforating Branches by Sex Across Intercostal Spaces.

		Female				Male				X^2^	*p*
		Present		Absent		Present		Absent			
		n	%	n	%	n	%	n	%		
1. ICS	Right	3	8.1	34	91.9	2	5.7	33	94.3	0.159	0.690 FDR *p* = 1.000 V = 0.047
	Left	13	35.1	24	64.9	5	14.3	30	85.7	4.170	0.041 * FDR *p* = 0.189 V = 0.241
2. ICS	Right	8	21.6	29	78.4	11	31.4	24	68.6	0.891	0.345 FDR *p* = 0.588 V = 0.111
	Left	6	16.2	31	83.8	12	34.3	23	65.7	3.132	0.77 FDR *p* = 0.192 V = 0.209
3. ICS	Right	9	24.3	28	75.7	12	34.3	23	65.7	0.864	0.353 FDR *p* = 0.588 V = 0.110
	Left	9	24.3	28	75.7	16	45.7	19	54.3	3.631	0.057 FDR *p* = 0.189 V = 0.225
4. ICS	Right	10	27	27	73	10	28.6	25	71.4	4.974	0.026 * FDR *p* = 1.000 V = 0.017
	Left	19	51.4	18	48.6	9	25.7	26	74.3	0.007	0.934 FDR *p* = 0.189 V = 0.263
5. ICS	Right	4	10.8	33	89.2	4	11.4	31	88.6	0.961	0.327 FDR *p* = 1.000 V = 0.010
	Left	6	16.2	31	83.8	3	8.6	32	91.4	3.309	0.069 FDR *p* = 0.686 V = 0.116

ICS: intercostal space, n: number of subjects, *: *p* < 0.05.

## Data Availability

The minimal dataset template is provided as Supplementary Materials. Full raw data supporting the findings of this study are available from the corresponding author upon reasonable request.

## Supplementary Materials

The following supporting information can be downloaded at: https://www.mdpi.com/article/10.3390/biomedicines14081863/s1, File S1: Ethics Committee Approval for the Study.
