# Supplementary material for: Comprehensive CT Angiography Assessment of Internal Thoracic Artery Morphometry, Anatomical Relationships, and Perforating Branches
Source: Biomedicines. 2026 Aug 20;14(8):1863. doi: 10.3390/biomedicines14081863 (PMC13509838; doi:10.3390/biomedicines14081863)
Supplement: Supplementary file 1 [file biomedicines-14-01863-s001.zip › biomedicines-4456593-supplementary File S1.pdf]

T.C.
SAKARYA ÜNİVERSİTESİ REKTÖRLÜĞÜ
Tıp Fakültesi Dekanlığı
Fakülte Girişimsel Olmayan Etik Kurulu

Sayı : E-71522473-050.01.04-300148 – 333 31.10.2023
Konu : Girişimsel Olmayan Etik Kurul Başvuru Dosyası

Sayın Doç. Dr. Keziban KARACAN

Sakarya Üniversitesi Tıp Fakültesi
Anatomi Anabilim Dalı

İlgi : 20.10.2023 tarih ve 333 sayılı başvurunuz.

Destekleyicisi olduğunuz **"Arteria thoracica interna'nın morfometrisinin bilgisayarlı tomografi ile değerlendirilmesi"** isimli çalışmanın ilgili belgeler araştırmanın gerekçe, amaç, yaklaşım ve yöntemleri dikkate alınarak incelenmiş olup; çalışmanın başvuru dosyasında belirtilen şekilde etik ve bilimsel açıdan sakınca bulunmadığına etik kurul üyelerince karar verilmiştir.

Ancak çalışma bitiş tarihinden itibaren en geç 3 ay içerisinde çalışma sonuç raporunun Girişimsel Olmayan Araştırmalar Etik Kuruluna gönderilmesi gerekmektedir.

Bilgilerinize rica ederim.

Prof. Dr. Ertuğrul GÜÇLÜ
Etik Kurulu Başkanı

Hasan KONAK
Etik Kurulu Sekr.

Güvenli Elektronik
İmzalı Aslı İle Aynıdır
31/10/2023

Bu belge, güvenli elektronik imza ile imzalanmıştır.

Doğrulama Kodu :BSEKJ5B6P5 Pin Kodu :10252 Belge Takip Adresi : https://turkiye.gov.tr/ebd?eK=5783&eD=BSEKJ5B6P5&eS=300148

Adres:Sakarya Üniversitesi Tıp Fakültesi Dekanlığı, Korucuk Kampüsü, Korucuk, Adapazarı/Sakarya
Telefon No:264 295 6630 Faks No:264 295 6629
e-Posta:tip@sakarya.edu.tr Elektronik Ağ:www.tip.sakarya.edu.tr
Bilgi için: Hasan Konak
Unvanı: Sürekli İşçi
